# P-984. A Novel Tool for Assessing Antibiotic Stewardship Programs in Inpatient Healthcare Facilities Across the Globe

**DOI:** 10.1093/ofid/ofaf695.1183

**Published:** 2026-01-11

**Authors:** Twisha S Patel, Payal K Patel, Valeria Fabre, Sara E Cosgrove, Rodolfo E Quirós, Vu Thi Lan Huong, H Rogier van Doorn, Raph L Hamers, Direk Limmathurotsakul, Abhilasha Karkey, Elizabeth Dodds Ashley, Deverick J Anderson, Julia E Szymczak, Ebbing Lautenbach, Keith W Hamilton, Naledi Mannathoko, Mosepele Mosepele, Marc Mendelson, Katharina Kranzer, Fernanda C Lessa

**Affiliations:** Centers for Disease Control and Prevention, Atlanta, GA; Intermountain Healthcare, Salt Lake City, UT; Johns Hopkins University School of Medicine, Baltimore, MD; Johns Hopkins School of Medicine, Baltimore, MD; CoNaCRA, CABA, Ciudad Autonoma de Buenos Aires, Argentina; Oxford University Clinical Research Unit, Ho Chi Minh, Ho Chi Minh, Vietnam; University of Oxford, Hanoi, Ha Noi, Vietnam; Oxford University Clinical Research Unit, Ho Chi Minh, Ho Chi Minh, Vietnam; Mahidol-Oxford Tropical Medicine Research Unit, Faculty of Tropical Medicine, Mahidol University, Bangkok, Thailand, Bangkok, Krung Thep, Thailand; Oxford university clinical research unit Nepal, Lalitpur, Bagmati, Nepal; Duke Center for Antimicrobial Stewardship and Infection Prevention, Durham, NC; Duke Center for Antimicrobial Stewardship and Infection Prevention, Durham, NC; University of Utah, Salt Lake City, Utah; University of Pennsylvania, Philadelphia, Pennsylvania; University of Pennsylvania Perelman School of Medicine, Philadelphia, Pennsylvania; University of Botswana, Gaborone, Southern, Botswana; University of Botswana, Gaborone, Southern, Botswana; University of Cape Town, Cape Town, Free State, South Africa; The Health Research Unit Zimbabwe, Biomedical Research and Training Institute, Harare, Zimbabwe, Harare, Harare, Zimbabwe; CDC, Atlanta, Georgia

## Abstract

**Background:**

Antibiotic stewardship (AS) practices vary widely across countries based on available infrastructure and resources. Thus, implementing AS in healthcare facilities (HCFs) across the globe cannot take a "one-size-fits-all" approach. In collaboration with Johns Hopkins University, University of Oxford with Duke University, and the University of Pennsylvania, the US CDC developed and validated a Global Antibiotic Stewardship Evaluation Tool (G-ASET) to help inpatient HCFs assess their AS practices and needs. Of note, the G-ASET is not intended to compare data across countries. An evaluation was performed using G-ASET to describe improvement opportunities in HCFs in Latin America, Asia, and Southern Africa.

**Figure 1.**
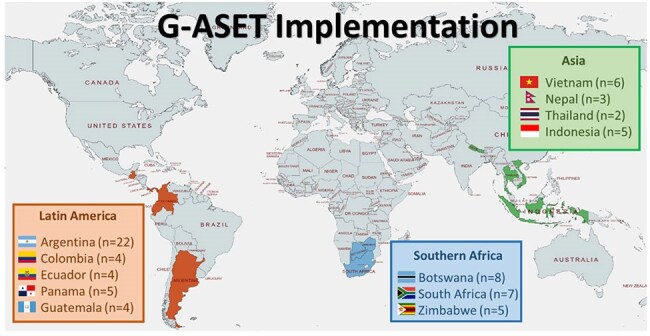
Distribution of the 75 Participating Inpatient Healthcare Facilities

**Figure 2.**
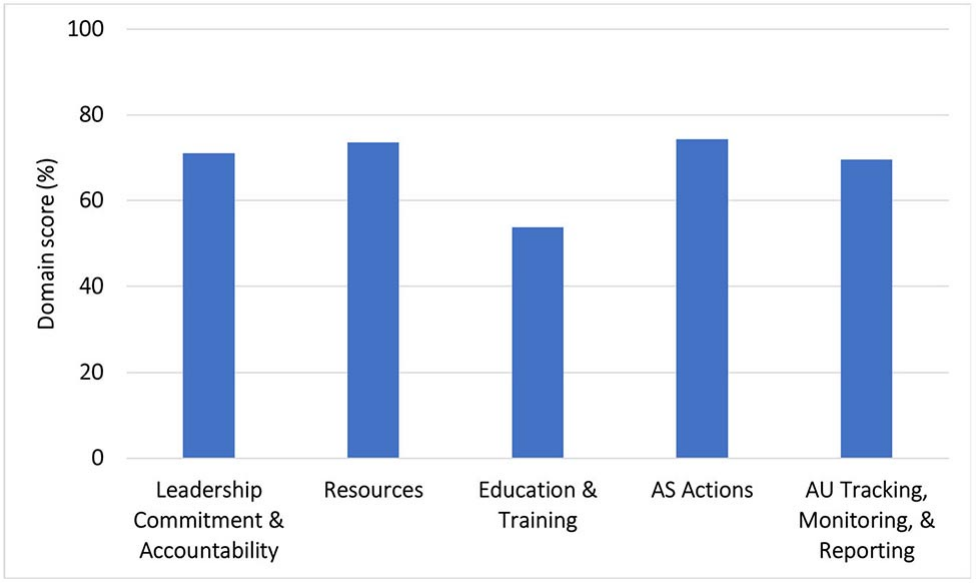
G-ASET Domain Scores (N=75 Participating Inpatient Healthcare Facilities)

**Methods:**

Between July 2021-December 2022, G-ASET was developed using a multi-step process that emphasized clinical relevancy and feasibility including literature evaluation, multiple rounds of revision by a multidisciplinary team of AS experts, and assembly of an expert consensus panel using a modified Delphi approach. Assessment items in G-ASET are organized using 5 domains: 1) leadership commitment & accountability, 2) resources, 3) education & training, 4) AS actions, 5) antibiotic use tracking, monitoring, & reporting. The tool was validated by pilot testing in 10 sites: Argentina (n=3), Vietnam (n=1), Botswana (n=2), US (n=4). Finally, we performed a cross-sectional evaluation of AS programs using G-ASET in inpatient HCFs across 12 low- and middle-income countries between January 2023-April 2024 (Figure 1). Convenience sampling was used to purposively select diverse facilities. Overall and domain scores were calculated by summing points earned and converting to a percentage of potential points. Descriptive statistics were used for analysis.

**Results:**

Seventy-five inpatient HCFs were included in this evaluation (Figure 1). Overall G-ASET scores ranged from 40.4-89.7% across the HCFs. Education and training was the lowest scoring domain (53.7%) whereas AS actions was the highest scoring domain (74.3%) (Figure 2).

**Conclusion:**

Significant variability in overall G-ASET scores was observed among participating inpatient HCFs. The newly launched G-ASET can help to identify specific opportunities for improvement among AS programs in inpatient HCFs across the globe.

**Disclosures:**

Payal K. Patel, MD, MPH, FIDSA, Cormedix: Advisor/Consultant Elizabeth Dodds Ashley, PharmD, MHS, HealthtrackRx: Advisor/Consultant|UpToDate, Inc.: Author Royalties Keith W. Hamilton, MD, BioMerieux: Grant/Research Support

